# Systemic antibiotic prescribing to paediatric outpatients in 5 European countries: a population-based cohort study

**DOI:** 10.1186/1471-2431-14-174

**Published:** 2014-07-05

**Authors:** Jakob Holstiege, Tania Schink, Mariam Molokhia, Giampiero Mazzaglia, Francesco Innocenti, Alessandro Oteri, Irene Bezemer, Elisabetta Poluzzi, Aurora Puccini, Sinna Pilgaard Ulrichsen, Miriam C Sturkenboom, Gianluca Trifirò, Edeltraut Garbe

**Affiliations:** 1 Leibniz Institute for Prevention Research and Epidemiology, BIPS, Achterstr. 30, 28359 Bremen, Germany; 2 NIHR Biomedical Research Centre at Guy's and St Thomas' NHS Foundation Trust and King's College London, Department of Primary Care and Public Health Sciences, Room 713, 7th Floor, Capital House Weston St, SE1 3QD London, UK; 3 Health search, Italian College of General Practitioners, Via Sestese, 61 - 50141 Florence, Italy; 4 Agenzia regionale di sanità della Toscana, Via Dazzi, 1 - 50141 Florence, Italy; 5 Department of Medical Informatics, Erasmus University Medical Center, Dr. Molewaterplein, 50 3015 GE Rotterdam, The Netherlands; 6 The PHARMO Institute, Van Deventerlaan 30-40, 3528 AE Utrecht, The Netherlands; 7 Department of Pharmacology, University of Bologna, Via Irnerio, 48, 40126 Bologna, Italy; 8 Department of Clinical Epidemiology, Aarhus University Hospital, Olof Palmes Allé 43-45, Aarhus, Denmark; 9 Department of Clinical and Experimental Medicine and Pharmacology, University of Messina, Messina, Italy

**Keywords:** Drug utilisation study, Antibiotic resistance, Paediatric, Prescription rate, Cephalosporins, Macrolides, Penicillins, Electronic healthcare database

## Abstract

**Background:**

To describe the utilisation of antibiotics in children and adolescents across 5 European countries based on the same drug utilisation measures and age groups. Special attention was given to age-group-specific distributions of antibiotic subgroups, since comparison in this regard between countries is lacking so far.

**Methods:**

Outpatient paediatric prescriptions of systemic antibiotics during the years 2005-2008 were analysed using health care databases from the UK, the Netherlands, Denmark, Italy and Germany. Annual antibiotic prescription rates per 1,000 person years were estimated for each database and stratified by age (≤4, 5-9, 10-14, 15-18 years). Age-group-specific distributions of antibiotic subgroups were calculated for 2008.

**Results:**

With 957 prescriptions per 1000 person years, the highest annual prescription rate in the year 2008 was found in the Italian region Emilia Romagna followed by Germany (561), the UK (555), Denmark (481) and the Netherlands (294). Seasonal peaks during winter months were most pronounced in countries with high utilisation. Age-group-specific use varied substantially between countries with regard to total prescribing and distributions of antibiotic subgroups. However, prescription rates were highest among children in the age group ≤4 years in all countries, predominantly due to high use of broad spectrum penicillins.

**Conclusions:**

Strong increases of antibiotic prescriptions in winter months in high utilising countries most likely result from frequent antibiotic treatment of mostly viral infections. This and strong variations of overall and age-group-specific distributions of antibiotic subgroups across countries, suggests that antibiotics are inappropriately used to a large extent.

## Background

Antibiotics are among the most widely prescribed medications in Europe [1]. Resistance to common antibiotic agents has grown among a majority of bacterial pathogens and is widely acknowledged to be an increasing threat to global public health [2,3]. Population exposure to antibiotics is recognised as an important cause for the emergence of resistant bacterial strains [4-6]. Due to a high burden of respiratory infections in paediatric populations, antibiotic prescribing is particularly common in the treatment of childhood diseases. However, frequent childhood respiratory conditions such as sore throat, acute otitis media, acute cough, sinusitis, common cold, and acute bronchitis are predominantly caused by viruses and mostly do not benefit from antibiotic therapy [7-10]. Thus, high prescribing of antibiotic agents to the paediatric population is a recognised indicator for inappropriate prescribing patterns in primary care [11].

Several studies have been published in the last decade either assessing antibiotic use in paediatric populations of single European countries [11-15] or conducting comparisons of paediatric antibiotic use between up to three countries [16,17]. Findings showed wide variations across Europe in the prescribing of systemic antibiotics to children and adolescents [12]. Comparability of these studies was, however, limited due to differences in drug utilisation measures, inclusion criteria of the study populations, age group categorizations and classifications of antibiotic subgroups [11-18]. In addition, comparison of the age-group-specific distributions of antibiotic subgroups between countries is lacking so far.

The aim of the present study was to compare outpatient prescribing of systemic antibiotics to children and adolescents in the age group 0-18 years between Denmark, Italy, Germany, the Netherlands and the UK for the years 2005-2008, based on a standardised protocol for data extraction and analysis for each database in these countries. Special attention was paid to differences of age-group-specific use of different antibiotic subgroups across countries. Seasonal variations of prescribing rates were described to assess impact of antibiotic treatment of mostly viral respiratory infections during winter months on total use.

## Methods

### Data sources

Data were retrieved from one general practice database (The Health Improvement Network (THIN), UK), one outpatient pharmacy dispensing database (PHARMO, the Netherlands) and three claims databases (Aarhus University Hospital Database, Denmark; German Pharmacoepidemiological Research Database (GePaRD), Germany; Emilia Romagna regional database, Italy). These electronic healthcare databases cover a total source population of about 23 million persons. All databases are in compliance with European Union guidelines on the usage of medical data for research. The study was given approval by regulatory agencies or by scientific and ethical advisory boards of the databases where applicable. All five databases comprise medical information of a defined population. Detailed descriptions of these databases including specifics regarding approvals for use of data for this study are enclosed as Additional file 1.

### Study design and statistical analysis

The study was conducted in an open (dynamic) cohort design. The study period was from January 2005 to December 2008, since for some databases no more recent data was available at the time of the analysis. The observational period of the Italian Emilia Romagna Database was restricted to the years 2007 and 2008, since data of the years 2005 and 2006 were not available. Cohort start was defined as January 1^st^ 2005 or – if later - the first date a person entered into the respective database. Cohort exit was defined as exit of the person from the database, 18^th^ birthday, death, the first interruption of follow-up in the database or December 31^st^ 2008, whichever came first.

Over the follow-up period, members of the study population could contribute to more than one age category. Children and adolescents up to the age of 18 years were included and divided into the age groups ≤4, 5-9, 10-14, and 15-18 years. This age group classification was chosen, since it was commonly used in other studies of antibiotic utilisation in the paediatric setting and hence allows comparison of age-group-specific use across studies [11,15,19,20].

Utilisation of systemic antibiotics (Anatomical Therapeutic Chemical (ATC) code: J01) was measured as the annual prescription rate, i.e. the number of prescriptions divided by 1,000 person years. Person years rather than individuals were used as denominator, given that not all children could be followed for an entire year. Prescription rates were chosen as a main outcome measure instead of Defined Daily Doses (DDDs) per person time, since dosing of antibiotics depends on a patient’s age and body weight. Prescription rates are therefore more appropriate to describe antibiotic use among children and conduct comparison between children in different age groups than DDDs per person time [12]. Seasonal trends were analysed by monthly prescription rates per 1,000 person years. To express utilisation on the level of chemical substances, the annual prescription rate per 1,000 person years, was estimated for single agents for the year 2008 as this was the year to which all databases contributed.

Outpatient prescriptions of systemic antibiotics were divided into the following subgroups (ATC codes in brackets): Tetracyclines (J01AA), broad spectrum penicillins (J01CA, J01CR), narrow spectrum penicillins (J01CE, J01CF), second generation cephalosporins (J01DC), third generation cephalosporins (J01DD), sulphonamides/trimethoprim (J01EB, J01EE, and J01EA), macrolides (J01FA) and nitrofuran derivatives (J01XE). Less frequent antibiotics were pooled in the subgroup ‘others’.

To describe differences in the distribution of antibiotic subgroups between countries, age-group-specific proportions of antibiotic subgroups were calculated for each database in the year 2008 based on the respective total number of systemic antibiotic prescriptions per age group.

Local data extraction was conducted by using standardised purpose-built Jerboa® software, which was previously developed by the Erasmus University Medical Center and tested against different scripts [21]. Measures of antibiotic utilisation as much as the corresponding numerators and denominators for each database population were calculated locally on different levels of the ATC Classification System, stratified by age in years, sex, calendar months and calendar year. These analyses followed a common protocol. Anonymised and aggregated data were sent to a remote research environment (RRE) at Erasmus University in Rotterdam, the Netherlands, which could be accessed via a secured password to conduct further statistical analyses. These further analyses were conducted using SAS® 9.2.

## Results

The average annual total population comprised 334,991 children from Denmark, 773,492 children from the Italian region Emilia Romagna, 1,340,163 children from Germany, 622,450 children from the Netherlands and 798,253 children from the UK.

With 957.2 prescriptions per 1,000 person years, the highest annual prescription rate in the year 2008 was found in Emilia Romagna (Italy) followed by Germany (560.8), UK (555.2), Denmark (481.0) and the Netherlands (294.2). This ranking did not change over the entire observational period, with the restriction, that data from Italy was only available for the years 2007 and 2008 (Table 1).

**Table 1:** Annual prescription rates per 1,000 person years of systemic antibiotics per age group in the years 2005-2008 (children and adolescents ≤18 years)

		**Aarhus (DK)**	**Emilia Romagna**^**a**^**(IT)**	**GePaRD (DE)**	**PHARMO (NL)**	**THIN ** **(UK)**
2005	≤4	800.0	.	984.7	519.4	891.5
	5-9	356.9	.	683.0	275.0	486.4
	10-14	264.0	.	441.1	147.2	385.4
	15-18	451.9	.	601.2	250.7	582.3
	0-18	467.7	.	664.6	296.9	575.2
2006	≤4	869.5	.	949.7	543.9	882.8
	5-9	384.7	.	656.9	288.1	478.0
	10-14	285.1	.	405.9	160.2	386.2
	15-18	497.3	.	565.7	267.8	581.0
	0-18	503.8	.	628.9	312.2	572.5
2007	≤4	977.1	1486.8	931.0	543.6	918.2
	5-9	386.9	1055.1	604.6	259.4	485.2
	10-14	285.0	661.0	378.8	151.6	391.1
	15-18	528.1	682.7	556.9	277.9	597.7
	0-18	531.1	1043.8	600.8	303.4	590.8
2008	≤4	981.8	1392.8	853.5	523.2	843.1
	5-9	325.1	982.8	560.4	256.3	450.9
	10-14	234.7	579.3	347.1	144.1	367.8
	15-18	504.4	610.2	561.5	274.5	581.1
	0-18	481.0	957.1	560.8	294.2	555.2

^a^Observational period of Emilia Romagna Database was available only for the years 2007 and 2008.

In all five countries and all years, the highest prescription rates were found in the age group ≤4 years and the lowest rates were observed in the age group 10-14 years (Table 1).

**Figure 1:**
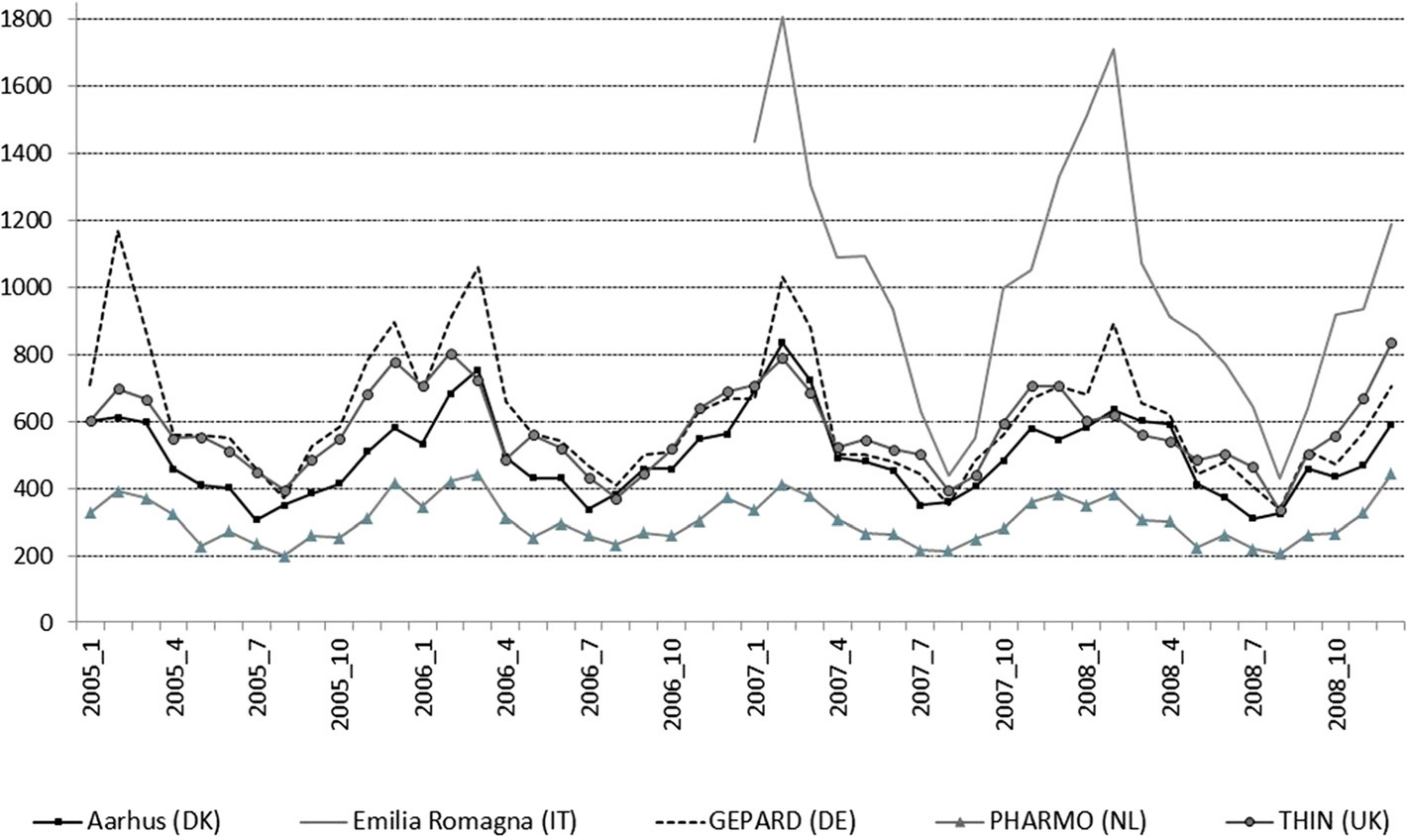
**Trends in monthly antibiotic prescription rates per 1,000 person years and country during the observed years (2005-2008)**^**a **^**in children and adolescents (≤18 years of age). **^a^Observational period of Emilia Romagna Database was available only for the years 2007 and 2008.

Prescription rates in children and adolescents in the Netherlands and the UK fluctuated slightly between the years 2005 and 2008, overall and in different age groups (Table 1). Similarly, the number of prescriptions per 1,000 person years in Danish children changed marginally throughout the course of the study. Nevertheless, an increase by 22.7% could be observed in the age group ≤4 years between 2005 and 2008 (Table 1). In Germany, a progressive decline of the annual prescription rates could be observed over all four age groups during the study period (Table 1).Monthly prescription rates were lowest in July and August and rose continuously until reaching their peak between December and March of the following year. Seasonal increases in the winter months were most pronounced in Italy followed by Germany (Figure 1).

In all countries except Denmark, broad-spectrum penicillins formed the largest subgroup of prescribed systemic antibiotics, with proportions varying between 23.8% in Germany and 57.4% in Italy (Table 2). Proportions of broad spectrum penicillins were highest in the age group ≤4 years and decreased gradually with age in all five countries. Narrow-spectrum penicillins were most widely used in Denmark (51.7%) and covered different proportions in the four other countries, from 0.1% (Italy) to 23.5% (UK) (Table 2).

**Table 2:** Distribution of systemic antibiotic subgroups^a^ by age group in 2008 (children and adolescents ≤18 years)

	**Aarhus (DK)**					**Emilia Romagna (IT)**					**GePaRD (DE)**					**PHARMO (NL)**					**THIN (UK)**				
	**≤ 4**	**5-9**	**10-14**	**15-18**	**0-18**	**≤ 4**	**5-9**	**10-14**	**15-18**	**0-18**	**≤ 4**	**5-9**	**10-14**	**15-18**	**0-18**	**≤ 4**	**5-9**	**10-14**	**15-18**	**0-18**	**≤ 4**	**5-9**	**10-14**	**15-18**	**0-18**
Tetracyclines	-	-	0.3	1.7	0.4	<0.1	<0.1	1.1	4.1	0.6	<0.1	0.1	3.8	11.7	3.3	<0.1	0.1	9.6	24.2	6.7	<0.1	<0.1	8.6	26.3	7.8
Broad spectrum penicillins	47.8	24.2	12.7	14.8	31.0	62.4	57.5	51.3	44.0	57.4	28.4	23.5	23.3	17.5	23.8	73.3	60.3	43.0	21.8	54.9	62.0	47.3	34.7	20.8	44.2
Narrow spectrum penicillins	42.6	63.8	69.4	48.5	51.7	<0.1	0.1	0.2	0.2	0.1	11.7	19.8	15.4	12.7	14.8	4.9	9.7	13.7	12.7	8.9	16.0	28.1	31.8	25.1	23.5
Second generation cephalosporins	<0.1	<0.1	<0.1	<0.1	<0.1	5.1	4.7	3.0	2.0	4.3	27.5	21.1	15.6	8.7	19.4	0.1	0.3	0.1	0.1	0.2	1.2	1.1	0.8	0.4	0.9
Third generation cephalosporins	-	-	-	<0.1	<0.1	14.7	14.8	13.9	10.6	14.1	8.8	5.9	5.2	3.8	6.3	<0.1	<0.1	0.1	0.2	0.1	0.1	0.1	0.1	<0.1	0.1
Sulfonamides/trimethoprim	0.9	2.8	3.5	9.5	3.6	0.4	0.5	0.7	1.0	0.5	3.8	5.4	5.5	7.8	5.4	5.3	7.1	6.3	6.5	6.1	4.9	6.4	5.2	7.7	5.9
Macrolides	8.3	7.6	13.0	24.7	12.5	16.7	21.0	27.2	28.9	20.7	16.7	18.8	24.9	23.5	20.3	15.2	17.4	18.7	14.2	16.0	12.2	11.7	13.7	12.6	12.5
Nitrofuran derivatives	0.1	1.4	0.8	0.7	0.6	<0.1	<0.1	<0.1	<0.1	<0.1	0.2	0.6	0.4	0.4	0.4	0.7	4.3	6.9	16.3	5.8	0.2	0.5	0.4	0.8	0.4
Others	0.2	0.2	0.3	0.3	0.2	0.6	1.4	2.8	9.1	2.1	2.8	4.7	5.9	13.9	6.3	0.5	0.9	1.8	3.9	1.4	3.4	4.8	4.7	6.3	4.6

^a^In column percentages, based on total number of systemic antibiotic prescriptions per age group.

Cephalosporins were hardly prescribed to Danish and Dutch children, whereas second and third generation cephalosporins were the most prescribed cephalosporins in Germany and Italy, respectively. Relative use of second and third generation cephalosporins was highest in the age group ≤4 years and gradually decreased with age (Table 2).

Use of macrolides increased with age and accounted for 20.7% of total use in Italy, 20.3% in Germany, 16.0% in the Netherlands, 12.5% in the UK and 13.5% in Denmark. Proportions of macrolide use increased with age (Table 2).

Overall, tetracyclines covered varying proportions, from 0.4% in Denmark to 7.8% in the UK. In line with age restrictions, relevant relative use of tetracyclines was only found above ten years of age in all five countries (Table 2).

Amoxicillin and clarithromycin were among the 12 agents with the highest annual prescription rates in all databases (Table 3). Amoxicillin was either the most or among the three most commonly prescribed agents. Only in Italy, Amoxicillin plus enzyme inhibitor showed the highest prescription rate. Phenoxymethylpenicillin (e.g. penicillin V) was most prescribed in Denmark and was also frequently prescribed in Germany and the UK. In contrast, this agent was not prescribed to Italian children and its use in the Netherlands was negligible (Table 3).

**Table 3:** Annual prescription rates per 1,000 person years of single antibiotic agents and combinations in 2008 (children and adolescents ≤18 years)

**Antibiotic agent**^**a**^	**Aarhus (DK)**	**Emilia Romagna (IT)**	**GePaRD (DE)**	**PHARMO (NL)**	**THIN (UK)**
***Tetracyclines***					
Doxycycline	0.2	1.2	10.6	11.9	6.3
Lymecycline	0.6	1.5	.	.	16.4
Oxytetracycline	<0.1	.	.	.	13.9
Minocycline	.	3.4	6.3	6.9	6.1
***Penicillines***					
Amoxicillin	121.5	230.8	120.7	124.1	221.0
Pivmecillinam	17.6	.	.	.	<0.1
Phenoxymethylpenicillin	219.1	.	70.5	0.3	71.9
Pheneticillin	-	-	-	15.9	-
Dicloxacillin	29.4	.	<0.1	.	.
Flucloxacillin	0.1	<0.1	0.4	9.7	58.2
Amoxicillin plus enzyme inhibitor	7.3	318.0	11.0	37.6	23.3
***Cephalosporines***					
Cefalexin	<0.1	0.7	1.2	<0.1	16.6
Cefadroxil	.	0.4	12.5	.	0.6
Cefuroxime	0.1	8.8	25.2	0.3	0.2
Cefaclor	.	27.9	81.2	0.2	5.0
Ceftriaxone	<0.1	9.8	0.2	0.1	<0.1
Cefixime	.	58.6	16.1	.	0.4
Cefpodoxime	.	43.5	16.0	.	<0.1
Ceftibuten	.	22.6	2.9	<0.1	.
***Sulfonamides and Trimethoprim***					
Trimethoprim	2.9	.	4.1	5.5	32.0
Sulfamethizole	14.2	.	.	.	.
Sulfamethoxazole and trimethoprim	.	5.1	26.2	12.4	0.8
***Macrolides***					
Erythromycin	27.0	1.6	36.8	4.0	56.8
Roxithromycin	7.1	3.2	16.1	0.1	.
Clarithromycin	5.4	96.7	26.5	16.9	8.5
Azithromycin	20.8	92.1	34.3	25.8	4.1
***Nitrofuran derivatives***					
Nitrofurantoin	2.8	<0.1	2.1	17.0	2.3
***Other antibacterials***					
Fosfomycin	.	9.2	0.6	0.2	.
***Others***	5.0	22.0	38.9	5.3	10.5

^a^The 12 most prescribed agents per database in 2008 were selected. Remaining agents were labelled as ‘others’.

## Discussion

Our study provides comprehensive information on the utilisation of systemic antibiotics among children and adolescents in the age group ≤18 years in Denmark, Italy, Germany, the Netherlands and the UK during the years 2005 to 2008. Our findings illustrate striking variations of total systemic antibiotic use in paediatric outpatient care between these countries. Substantial differences of outpatient antibiotic use among children across Europe have been described before, but these previous studies only provided comparable data of drug use for up to three countries and suffered from different definitions of drug utilisation measures. Furthermore, comprehensive data about age-group-specific distributions of antibiotic subgroups was lacking for most countries of this study and a comparison has not been conducted so far. In 2001, the European Surveillance of Antimicrobial Consumption Project was established to gather reliable and comparable information on the utilisation of antibiotics in Europe, however, without distinguishing between adults and children [1]. The current study captured outpatient systemic paediatric antibiotic use of five countries in different European regions ensuring high inter-country comparability, due to consistent definition of drug utilisation measures, age groups and classification of antibiotic subgroups.

Overall, the annual antibiotic prescription rates in the Italian region Emilia Romagna were more than three times higher than those in the Netherlands, the country with the lowest prescription rates, and still substantially higher than those in Germany, the country with the second highest use. When compared to other studies, magnitude of paediatric antibiotic use in Italy exceeded use reported for Canada (608 prescriptions per 1000 children <15 years of age in 2003) [19] and Sweden (764 prescriptions per 1000 children 0-6 years of age in 2002) [15] as well, but appears to be comparable to the U.S. (910 prescriptions per 1000 person years in children <18 years of age in 2001) [22].

High antibiotic prescribing in the Italian outpatient setting compared to the other countries in our study might be related to differences with regard to historical backgrounds, cultural and social factors, awareness about antibiotic resistance in the community and among healthcare providers [23] as well as the ability of physicians to adequately diagnose common infectious diseases [16]. So far, reasons for strong variations of antibiotic use across European countries have not yet been fully investigated. Nevertheless, previous studies suggest that awareness about antibiotic resistance [24] and inadequacy of antibiotics to treat viral infections [25] is poor among Italian patients and perception of parent expectations by Italian physicians is a major determinant of antibiotic prescribing to children [26].

In contrast, several previous studies showed antibiotic utilisation in the Netherlands to be lowest in Europe, overall and in the paediatric setting [6,13]. The Netherlands are a country with a strict prescribing policy for antiinfectives, and there are intensive efforts into promoting guideline-appropriate prescribing habits to combat antibiotic resistance [27].

Although antibiotic use was by far the highest among Italian children and adolescents, antibiotic prescription rates in Denmark, Germany and UK still exceeded those in the Netherlands to a great extent. These observed strong variations of total paediatric antibiotic use among the countries of study are unlikely to reflect an actual therapeutic need which would have to be based on marked differences in the burden of infectious diseases between these countries. This assumption is also supported by the observed pronounced increases of prescription rates during winter months which were expectedly highest in Italy, and smallest in the Netherlands. Increases of antibiotic use are most likely related to seasonal rise of predominantly viral respiratory infections and hence should be limited [1].

Since our findings could not provide information beyond the 4-year study period, we compared our prescription rates with those of other studies which included other study years or longer time periods. In this respect, our data for the years 2005-2008 in the Netherlands agreed well with the findings by de Jong et al. who reported a variation of the total annual number of antibiotic prescriptions between 282 and 307 per 1,000 Dutch children in the years 1999-2005 [13]. This suggests an overall stable total antibiotic use among Dutch children for almost ten years. Gagliotti et al. observed annual prescription rates per 1,000 person years among children 0-14 years of age from Emilia Romagna, varying between 1,158 and 1,358 during 2000-2002 [13]. This is in line with our findings in children below 15 years of age of 1,123 (2007) and 1,034 prescriptions per 1,000 person years (2008), indicating marginal changes over time of total paediatric use in Emilia Romagna. Prescription rates among British children did not show any apparent trend towards lower or higher prescribing in our study over the study years. Gradual annual increases of prescription rates between 2000 and 2007 were reported in the UK based on data from the General Practice Research Database (GPRD) [11]. However, differences to our findings for the years 2005 to 2007 were small and might have resulted from variations in the regional distribution of general practices contributing data to THIN and/or the GPRD. We observed a steady decrease in prescription rates in Germany during 2005-2008. Another German study also based on GePaRD data found slightly higher prescription rates among German children without an obvious downward trend for the years 2004-2006 [18]. This former study, however, included data from four rather than three health insurances, resulting in a study population of about twice as many children as in this study which may explain the difference.

We also detected remarkable differences in the choice of antibiotic subgroups between the countries of our study. Narrow spectrum penicillins formed the majority of systemic antibiotics in Denmark, whereas prescriptions of broad spectrum penicillins were most frequent in the four other countries. In line with that, the highest agent-specific prescription rates were reported for phenoxymethylpenicillin in Denmark, amoxicillin in Germany, the Netherlands and the UK and amoxicillin plus enzyme inhibitor in Italy. Relatively high use of narrow spectrum penicillins in Denmark in comparison to other European countries has also been reported previously [6]. However, it is noteworthy that even though proportions for narrow spectrum penicillins were highest in Denmark, broad spectrum penicillins formed the antibiotic subgroup most frequently prescribed to children in the age group ≤4 years in all 5 countries. This might be due to frequent use of amoxicillin or amoxicillin and enzyme inhibitor in the treatment of acute otitis media, which shows the highest incidence in the first two years of life [28].

Macrolides were commonly prescribed in all five countries with the highest use in the age groups 10-14 and 15-18 years. Relative proportions of macrolide use were lowest in Denmark. This finding is in agreement with a Danish practice guideline which recommends restricting the use of macrolides to patients with penicillin allergies in the treatment of common childhood infections [21]. Several studies from the U.S. and Europe show a strong association of high macrolide use and the emergence of resistant strains of pneumococci and other common pathogens [23-25]. Hence high prescription rates of macrolides are questionable and likely to unnecessarily increase selective pressure on bacterial pathogens. In particular high use of clarithromycin and azithromycin in the Emilia Romagna region appears unjustified, since international guidelines do not recommended these agents as first-line treatment of common childhood infections [29-32]. Furthermore, longer plasma half-life of azithromycin and clarithromycin in contrast to erythromycin might even accelerate the emergence of antibiotic resistance [33,34].

Our findings regarding paediatric cephalosporin use are in line with previous studies which reported strong variations of cephalosporin prescribing across Europe, with the lowest prescription rates in the Netherlands and Denmark [13,18-20,35]. Overall, the prescription rate of cefaclor (a second generation cephalosporin) in German children was the second highest after amoxicillin, and use of second generation cephalosporins was particularly common in very young children. Only in Italy, the parenterally administered third generation agent ceftriaxone was prescribed frequently. Considerably higher prescribing of parenteral antibiotics in Italian outpatient care in contrast to Northern European countries has been reported previously [36]. The high relative use of cephalosprines in Germany and Italy as observed here, suggests frequent prescribing of these antibiotics as a first–line treatment of common paediatric respiratory infections. This is in conflict with international practice guidelines [29,30] recommending that cephalosporins should be preserved for second-line treatment in cases such as treatment failure of first-line agents, non-type 1 allergy to penicillins or unusually severe symptoms.

### Strengths and limitations

Our study overcomes limitations of previous studies and facilitates the comparison of paediatric antibiotic prescriptions in five countries based on a common protocol using the same drug utilisation measures. It provides insight into the age-group-specific distributions of antibiotic subgroups in the paediatric setting of the participating countries. Ascertainment of antibiotics prescribed in the outpatient setting was complete in all databases except Denmark, where some antibiotics as e.g. cephalosporins are reimbursable only in particular circumstances and might therefore have been underascertained. Nevertheless, given that the Danish National Health System reimburses antibiotics for the entire spectrum of childhood indications, [37] the proportion of antibiotics which could not be captured due to private prescribing appears to be small. Besides this, differences of antibiotic use across countries reflect differences in prescribing behaviour of outpatient providers and not in the type of data.

Our study has some limitations, which have to be taken into consideration. First, for this study only data for the years 2007 and 2008 was available from the Northern Italian region Emilia Romagna. Hence, insight into the development of antibiotic prescribing over time is limited. However, our findings are in good agreement with Gagliotti et al. [14] In addition, extrapolation from our findings to Italy in general is not straight forward, given considerable regional differences of prescribing patterns in Italy. Nonetheless, previous studies about marked heterogeneity of antibiotic use across Italy with up to 19% higher paediatric prevalence rates of antibiotic exposure in southern regions compared to Emilia Romagna [35] indicate, that overall paediatric antibiotic use in the Italian outpatient setting during the years of our study might have been even higher than suggested by our findings.

Since all five databases only provide information on drugs prescribed in the outpatient setting, antibiotics administered to inpatients to treat severe childhood infections could not be studied. Given that indications underlying the issued prescriptions were not available in all databases, the appropriateness of single treatment courses could not be assessed. Additionally, compliance with the antibiotic prescription remains unknown.

## Conclusions

Comparison of paediatric antibiotic consumption between different European countries revealed a wide variability of antibiotic prescribing patterns. Strong variations of overall and age-group-specific distributions of antibiotic subgroups across countries, suggests that antibiotics are inappropriately used to a large extent. Considerably higher prescription rates along with higher seasonal increases, particularly in Italy, in contrast to the Netherlands suggest frequent utilisation of antibiotics in the treatment of mostly viral respiratory infections. This study showed the benefit of using a common methodological approach to provide comparable and detailed data on paediatric antibiotic prescribing across Europe. Study results allow health care practitioners and policy makers to audit country and age-group-specific patterns of paediatric antibiotic use with regard to both total level of prescribing and the distribution of antibiotic subgroups/substances.

## Abbreviations

ATC: Anatomical therapeutic chemical classification system; DDD: Defined daily dose; GePaRD: German Pharmacoepidemiological Research Database; GP: General practitioner/family physician; GPRD: General Practice Research Database; THIN: The Health Improvement Network; RRE: Remote research environment; SHI: Statutory Health Insurance.

## Competing interests

JH, TS, GM, FI, AO, EP, AP, SPU and GT declare that they have no competing interest. IB has received grants from several pharmaceutical companies and funding organizations in the previous 3 years. MCS has received grants from Pfizer, grants from Boehringer, grants from Novartis and grants from Eli Lilly in the previous 3 years. MM has received grants from the International Serious Adverse Events Consortium, i SAEC (collaboration of academia and industry) in the previous 3 years. EG is running a department that occasionally performs studies for pharmaceutical industries. These companies include Bayer, Celgene, GlaxoSmithKline, Mundipharma, Novartis, Sanofi-Aventis, Sanofi Pasteur MDS, and STADA. EG has been a consultant to Bayer-Schering, Nycomed, GlaxoSmithKline, Teva and Novartis. EG is a member of the German Standing Vaccination Committee (Ständige Impfkommission, STIKO).

## Authors’ contributions

JH conceived and designed the study, conducted data analysis, drafted the article and had final approval. TS helped acquire the data and interpret the results and revise the article for content, and gave final approval. MM was involved in conception and design of the Study, made revisions to article drafts, and gave final approval for Publication. GM was involved with data acquisition and conception of the study, revised the article, and gave final approval for publication. FI helped design the study, helped with data acquisition and interpretation, and made revisions to article drafts, and gave final approval for publication. AO helped acquire the data and interpret the results and revise the article for content, and gave final approval. IB was involved with data acquisition, helped revise the article, and gave final approval for publication. EP helped acquire the data and interpret the results, and made revisions to article drafts, and gave final approval. AP was involved with data acquisition, helped revise the article, and gave final approval for publication. SPU helped acquire the data and interpret the results, and made revisions to article drafts, and gave final approval. MCS was involved in conception and design of the study, was involved with data acquisition and interpretation, helped revise the article, and gave final approval for publication. GT was involved in conception and design of the study, was involved with data acquisition and interpretation, helped revise the article, and gave final approval for publication. EG helped design the study, and acquire and interpret the data, supervised the publication and made substantial revisions to the article drafts, and gave final approval for publication. All authors read and approved the final manuscript.

## Pre-publication history

The pre-publication history for this paper can be accessed here:

http://www.biomedcentral.com/1471-2431/14/174/prepub

## Supplementary Material

Additional file 1 **Healthcare databases.** Additional file 1 provides a description of relevant characteristics of included healthcare databases. Click here for file
