# Supplementary material for: Systemic antibiotic prescribing to paediatric outpatients in 5 European countries: a population-based cohort study
Source: BMC Pediatr. 2014 Jul 5;14:174. doi: 10.1186/1471-2431-14-174 (PMC4099488; doi:10.1186/1471-2431-14-174)
Supplement: Additional file 1 — Healthcare databases. Additional file 1 provides a description of relevant characteristics of included healthcare databases. [file 1471-2431-14-174-S1.pdf]

Healthcare databases

The following is a description of relevant database characteristics. The supplied references provide in-depth descriptions.

Denmark

Data from Denmark was obtained from the population-based Aarhus University Hospital Database which links information of different medical electronic databases via unique pseudonymised person identification numbers. The database covers the entire population of the Northern and Central region of Jutland (~1.8 million Danish inhabitants), which accounts for about 30% of the total Danish population and includes information on demographics, outpatient dispensations reimbursed by the Danish National Healthcare Service including dates of outpatient visits, inpatient diagnoses and inpatient procedures. Outpatient prescription data comprise the date of prescribing, number of prescribed packages, defined daily doses (DDDs) per prescription and the commercial name of the drug. The database is maintained by the Department of Clinical Epidemiology, Aarhus University Hospital [1].

Access to data of the Aarhus University Hospital Database is not freely available. Use of data for this study was approved by the Danish Data Protection Agency (2006-53-1346). Given that this study did not involve human participants, prior ethical approval was not required.

Emilia Romagna, Italy

The data from Italy was gathered from the Emilia Romagna regional database which contains information on all health care services reimbursable by the Italian National Health Service for the about 4.5 million inhabitants of the Emilia Romagna region in Northern Italy. Information comprises demographic characteristics, outpatient

dispensations including the date of prescribing and the number of prescribed packages, inpatient diagnoses as well as inpatient diagnostic and surgical procedures. Prescription data can be linked to a drug register which contains information on the commercial name of the drug, the quantity of active substance of the drug contained in one package, DDDs of the active substance, and the estimated coverage of one package [2].

Access to Emilia Romagna data is not freely available. Use of data for this study was approved by Drug Policy Service of Emilia Romagna Authority. The study protocol was notified to the University Ethical Committee. Given that this study did not involve human participants, prior ethical approval was not required.

Germany

Data from Germany was gathered from the German Pharmacoepidemiological Research Database (GePaRD) that includes claims data from four German statutory health insurances (SHIs). GePaRD contains demographic information, information on hospitalisations, ambulatory physician visits including outpatient diagnoses and ambulatory dispensations of prescribed pharmaceuticals for more than 17 million insurants from all German federal states, covering over 20% of the total German population. Outpatient prescription data comprises prescribed quantities (number of packages), the prescription and the dispensation date. Preliminary analyses regarding age and sex distribution, the occurrence of different ICD-10-Groups in hospital discharge diagnoses and the frequency of drug dispensations of selected medications have shown the database to be representative for Germany [3,4].

Access to GePaRD data is not freely available. Three of the four SHIs contributing data to GePaRD approved the study, resulting in a source population of about 8 million insurants. In addition use of data for this study was approved by the Federal

Ministry of Health (for data from multiple federal states) and the health authority of Bremen (for data from the Federal State of Bremen). Given that this study did not involve human participants, prior ethical approval was not required.

Netherlands

Data from the Netherlands was retrieved from the PHARMO Database Network which covers 3.2 million inhabitants of 65 municipal areas in the Netherlands. The linked database network includes demographic and healthcare information from different settings [19]. For the current study, outpatient pharmacy dispensing data was used. This includes the date of dispensation, the dispensed quantity (number of units), type of unit (fluid, tablets etc.), and DDDs per prescription.

Representativity of PHARMO for the Dutch population has been shown previously [5].

Access to PHARMO data is not freely available. Ethical approval was not relevant because all data sources used are anonymous and are linked through probabilistic linkage using demographic variables of the patients. All other identifying information is deleted after the linkage of the various databases. Confidentiality of patient records will be maintained at all times. This approach is approved by the Dutch Data Protection Authority ('College Bescherming Persoonsgegevens').

UK

Data from the UK was retrieved from The Health Improvement Network (THIN) database which includes information from primary care medical records from over 500 general practices of about 5.9 million active registered patients. In the UK, all inhabitants have to be registered with a general practitioner (GP). THIN contains data including demographics, medical diagnoses and electronic prescriptions

automatically captured during consultations with GPs, specialist referrals and hospital admissions as well as some results of laboratory tests and lifestyle characteristics [6]. Practices contributing data are broadly representative of practices in the UK for patients' characteristics. Recording of consultations and prescriptions is comparable to national statistics [7] and the validity of the data for pharmacoepidemiological research has been established [6].

Access to THIN data is not freely available. Use of data for this study was approved by the EPIC Scientific Review Committee. Given that this study did not involve human participants, prior ethical approval was not required.

Reference List

1. Herings RM, Pedersen L: **Pharmacy-based Medical Record Linkage Systems.** In *Pharmacoepidemiology*. 5th edition, Editied by Strom B, Kimmel S, Hennessey S, Chichester: Wiley; 2012: 270-286.

2. Louis D, Yuen E, Maio V, Rabinowitz C, Robeson M, Smith K, Gonnella J: **A population based lognitudinal helathcare database in the Emilia Romagna Region, Italy: a resource for planning and research.** *Health Policy Newsletter* 2005, 18

3. Pigeot I, Ahrens W: **Establishment of a pharmacoepidemiological database in Germany: methodological potential, scientific value and practical limitations.** *Pharmacoepidemiol Drug Saf* 2008, 17:215-23.

4. Schink T, Behr S, Garbe E: **Externe Validierung von Verschreibungsdaten nichtsteroidaler Antirheumatika anhand des Arzneiverordnungs-Reports.** 54 Jahrestagung der Deutschen Gesellschaft für Medizinische Informatik, Biometrie und Epidemiologie (gmds) 2009.

5. Herings RM: **The PHARMO System.** *CNS Spectr* 2007, 9 (Suppl 16).

6. Denburg MR, Haynes K, Shults J, Lewis JD, Leonard MB: **Validation of The Health Improvement Network (THIN) database for epidemiologic studies of chronic kidney disease**. *Pharmacoepidemiol Drug Saf* 2011, 20:1138-49.

7. Raine R, Wong W, Ambler G, Hardoon S, Petersen I, Morris R, Bartley M, Blane D: **Sociodemographic variations in the contribution of secondary drug prevention to stroke survival at middle and older ages: cohort study.** *BMJ* 2009, 338:b1279.
